# The lateral femoral notch sign: a reliable diagnostic measurement in acute anterior cruciate ligament injury

**DOI:** 10.1007/s00167-018-5214-x

**Published:** 2018-10-13

**Authors:** Prabath C. A. M. Lodewijks, Diyar Delawi, Thomas L. Bollen, Gawein R. Dijkhuis, Nienke Wolterbeek, Jacco A. C. Zijl

**Affiliations:** 1 Department of Orthopaedic Surgery, St. Antonius Hospital, Koekoekslaan 1, 3435 CM Nieuwegein, The Netherlands; 2 Department of Rehabilitation, Physical Therapy Science and Sports, University Medical Center Utrecht, Heidelberglaan 100, 3584 CX Utrecht, The Netherlands; 3 Department of Radiology, St. Antonius Hospital, Koekoekslaan 1, 3435 CM Nieuwegein, The Netherlands

**Keywords:** Knee, ACL, General sports trauma, Imaging, Radiology

## Abstract

**Purpose:**

To describe the validity and inter- and intra-observer reliability of the lateral femoral notch sign (LFNS) as measured on conventional radiographs for diagnosing acute anterior cruciate ligament (ACL) injury.

**Methods:**

Patients (≤ 45 years) with a traumatic knee injury who underwent knee arthroscopy and had preoperative radiographs were retrospectively screened for this case–control study. Included patients were assigned to the ACL injury group (*n* = 65) or the control group (*n* = 53) based on the arthroscopic findings. All radiographs were evaluated for the presence, depth and location of the LFNS by four physicians who were blind to the conditions. To calculate intra-observer reliability, each observer re-assessed 25% of the radiographs at a 4-week interval.

**Results:**

The depth of the LFNS was significantly greater in ACL-injured patients than in controls [median 0.8 mm (0–3.1 mm) versus 0.0 mm (0–1.4 mm), respectively; *p* = 0.008]. The inter- and intra-observer reliabilities of the LFNS depth were 0.93 and 0.96, respectively. Secondary knee pathology (i.e., lateral meniscal injury) in ACL-injured patients was correlated with a deeper LFNS [median 1.1 mm (0–2.6 mm) versus 0.6 mm (0–3.1 mm), *p* = 0.012]. Using a cut-off value of 1 mm for the LFNS depth, a positive predictive value of 96% was found.

**Conclusion:**

This was the first study to investigate the inter- and intra-observer agreement of the depth and location of the LFNS. The depth of the LFNS had a very high predictive value for ACL-injured patients and could be used in the emergency department without any additional cost. A depth of > 1.0 mm was a good predictor for ACL injury. Measuring the depth of the LFNS is a simple and clinically relevant tool for diagnosing ACL injury in the acute setting and should be used by clinicians in patients with acute knee trauma.

**Level of evidence:**

Diagnostic study, level II.

## Introduction

Diagnosis of anterior cruciate ligament (ACL) injury is challenging in the acute setting. Swelling and pain can lower the reliability of clinical examination [7, 14], and MRI is often not directly available. Misdiagnoses could lead to delays in treatment that may further damage the knee joint. The lateral femoral notch sign (LFNS) has been described as a sign of chronically ACL-deficient knees on conventional radiographs. In chronic injuries, it is defined as a depression more than 1.5 mm deep in the lateral femoral condyle, near the terminal sulcus [4, 5, 10, 12, 18, 19]. In addition to the depth of the LFNS, the location might also be important for diagnosing ACL injury [5]. The origin of the LFNS was previously thought to be caused by repeated pivoting trauma due to chronic instability. More recent studies have described the presence of the LFNS in the acute setting [4, 5, 9, 13, 16, 18]. Nevertheless, the prevalence of a deep LFNS in the acute setting and the reliability of its measurement are not known. Furthermore, there is no clear cut-off point for depth of the impression that is associated with ACL ruptures in the acute setting. Several studies have used various cut-off points [4, 5, 9, 10, 16, 18, 23]. Due to the simplicity of the measurement, it could be a useful tool for the diagnosis of acute ACL injury, since conventional radiographs are often obtained in the acute setting to rule-out fractures. However, a clear definition regarding the depth of the LFNS with its associated prevalence in patients with acute ACL injuries needs to be obtained first. Furthermore, the reliability of the measurement needs to be evaluated. In this paper, the LFNS was evaluated as a valuable diagnostic tool for ACL injury in the acute setting by assessing the predictive value and the inter- and intra-observer reliability.

The purpose of this study was to describe the validity and reliability of the LFNS depth measurement in conventional radiographs. It was hypothesized that this measurement could be of additional value in diagnosing ACL injury in the acute setting.

## Materials and methods

In this case–control study, all patients with traumatic knee injury who underwent knee arthroscopy at our institution between May and December 2013 were evaluated. The inclusion criteria were that patients must be between the age of 12 and 45 years and that preoperative radiographs must be available. The exclusion criteria were: known previous knee fractures, previous ACL rupture, osteoarthritis, or a delay of more than 4 months between injury and imaging (to exclude chronic ACL injuries). The patients included in the study were assigned to the ACL injury group or the control group (no ACL injury), depending on arthroscopic findings. All radiographs were evaluated for the presence, depth and location of the lateral femoral notch sign by 4 blinded physicians that were involved in the diagnosis of the ACL injuries. Blinding was accomplished using a digital PACS viewer; the physicians were only able to see the relevant anonymous radiographs and had no access to additional patient information or medical history. Furthermore, retrospectively, information regarding the arthroscopy, including the presence of chondral defects, meniscal injuries and ligament ruptures, were collected from patient files. Baseline characteristics including gender, age, sport, and clinical examination were also retrieved.

### Radiological measurements

**Fig. 1:**
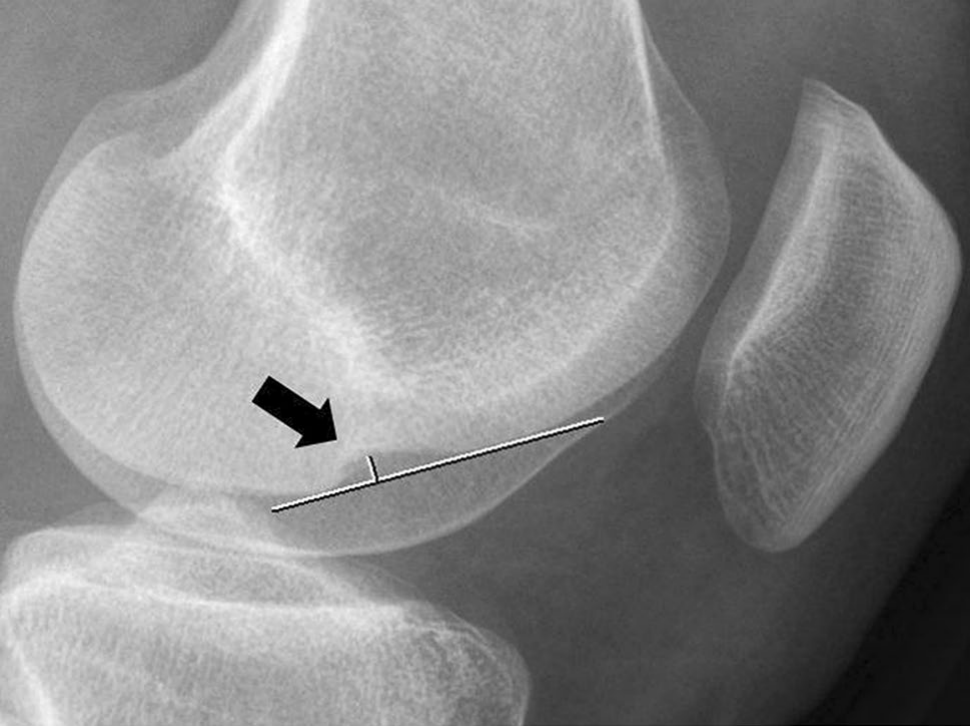
ACL-deficient knee with a deep lateral femoral notch sign. The depth was measured by drawing a line over the femoral condyle (long line) and a small line perpendicular to this line and the deepest point of the depression (black arrow)

**Fig. 2:**
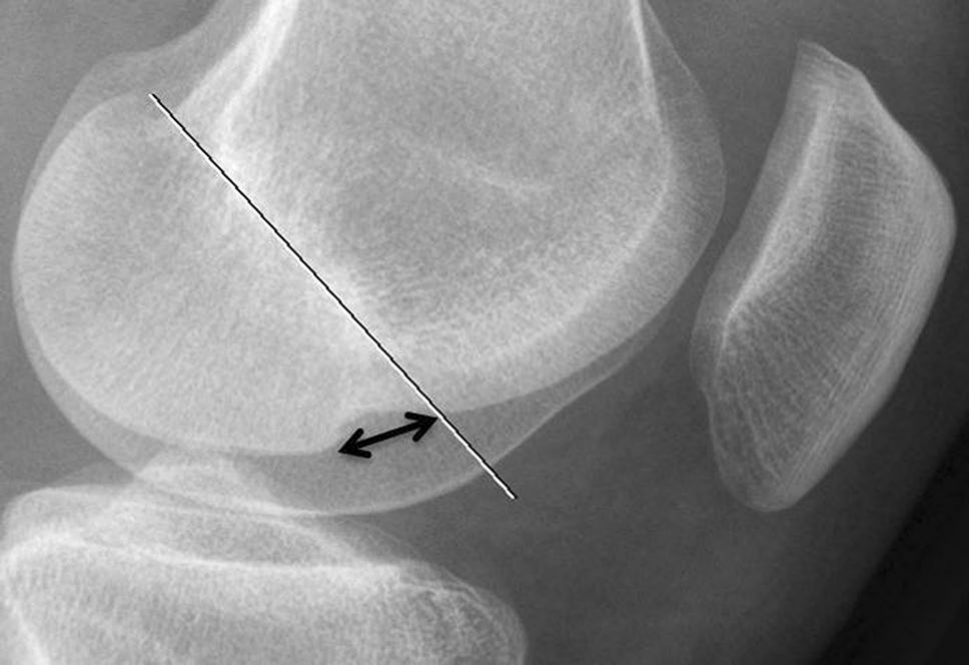
ACL-deficient knee illustrating the measured location for the lateral femoral notch sign (LFNS). The white line is the Blumensaat line. The black double-headed arrow (tangent line) is the distance between the Blumensaat line and the posterior beginning of the LFNS

All lateral radiographs of the knees were made using digital X-ray machines under standard operating procedures, resulting in radiographs with equal magnification factors. The LFNS was measured using a digital PACS viewer (Fig. 1). A tangent line was drawn from the normal articular surface of the lateral femoral condyle. A line from the tangent line to the deepest point of the lateral femoral notch gave the depth of the LFNS [1, 9, 10]. Based on the literature, the LFNS was divided into four categories, namely depths of 0.0 mm, 0.1–1.0 mm, 1.1–1.4 mm, and ≥ 1.5 mm [4, 5, 9, 10, 16, 18, 23]. The first two categories were considered normal depths, and the last 2 were considered pathological categories. To determine the location of the LFNS, the Blumensaat line was used [8, 11]. The Blumensaat line is a line through the roof of the intercondylar notch of the femur seen on the lateral radiograph of the knee. To determine the exact location, a tangent line to the lateral femoral condyle was drawn from the posterior beginning of the LFNS to the Blumensaat line [8]. A normal terminal sulcus does not extend more than 10 mm posterior to the Blumensaat line [5]. The location between the LFNS and the Blumensaat line was divided into three categories: 0 mm, 1–10 mm and > 10 mm (Fig. 2) [18]. These categories were chosen, based on the literature, to differentiate between normal and pathological locations [5, 8]. In this study, the depth and the location of the LFNS were combined to determine if a pooled measurement resulted in a more accurate prediction of ACL injury than isolated measurements of depth and location. Furthermore, the incidence of lateral meniscal injury and its correlation with the depth of the LFNS was studied [3, 9, 10, 13, 16, 27].

### Inter- and intra-observer reliability

All radiographs were evaluated by four specialists, consisting of two radiologists, an orthopaedic surgeon and a sports medicine physician. To calculate intra-observer reliability, each observer re-assessed 25% of the radiographs at a 4-week interval to prevent recall bias. The observers measured the depth of the LFNS within tenths of a mm and the location of the LFNS using its location relative to Blumensaat line in mm. All assessors were blinded to the characteristics and diagnoses of the patients and had no information about the arthroscopy. The radiographs were assessed on a high-contrast computer screen with a picture archive and communication system viewer. This retrospective study did not have to be reviewed by a medical ethics board according to the Dutch Law on Medical Research with Humans (WMO).

### Statistical analysis

Statistical analyses were performed using the statistical program SPSS^®^ (Statistical Package for the Social Sciences, Chicago, IL, Version 22.0). For differences between ACL-injured and non-ACL-injured knees, the Mann–Whitney *U* test was used for continuous data, and Fisher’s exact test was used for categorical data. According to general recommendations for evaluating measurement properties, at least 50 patients needed to be included [24]. Therefore, the aim was to include at least 100 patients.

Inter- and intra-observer agreement was measured using the intraclass correlation coefficient (ICC) with a two-way random effects model, single measurements and absolute agreement [22]. In general, an ICC of 0.70 is recommended as a minimum standard for test–retest reliability; an ICC of less than 0.40 is described as weak, between 0.41 and 0.60 is described as moderate, between 0.61 and 0.80 is described as substantial and > 0.81 is described as almost perfect agreement [25]. The 95% confidence interval (CI), the range of values containing the 95% confidence (the “true” correlation coefficient), was also calculated for the ICC.

Correlations in the ACL-injured group were measured with Spearman’s rho. Logistic regression analysis was used to find associations between the depth of the LFNS and other intra-articular injury, such as meniscal tears and chondral defects.

## Results

118 patients were eligible for this study, including 65 patients with ACL injury and 53 patients without ACL injury (control group) (Table 1). The ACL-injured group was younger, had more sport-related trauma and had a lower percentage of medial meniscal injuries. The sport-related trauma was mostly in high-risk pivoting sports (i.e., soccer, tennis, and basketball) [9].

**Table 1:** Patient characteristics and the location and depth measurements of the LFNS (mm)

	ACL injury (*n* = 65)	Controls (*n* = 53)	*p* value
Male gender (%)	43 (66.2%)	32 (60.4%)	n.s.
Mean age in years (SD)	25.4 (± 6.7)	32.5 (± 9.2)	< 0.001
Sport trauma	57 (87.7%)	9 (17.0%)	< 0.001
Football	30 (46.2%)	3 (5.7%)	
Hockey	9 (13.8%)	3 (5.7%)	
Tennis	1 (1.5%)	1 (1.9%)	
Other	17 (26.2%)	2 (3.8%)	
Medial meniscal injury	15 (23.1%)	36 (67.9%)	< 0.001
Lateral meniscal injury	26 (40.0%)	12 (22.6%)	n.s
Depth of LFNS			
Median (range)	0.8 mm (0–3.1 mm)	0.0 mm (0–1.4 mm)	0.008
Category			
0.0 mm	9 (13.8%)	27 (50.9%)	< 0.001
0.1–1.0 mm	32 (49.2%)	25 (47.2%)	
1.1–1.4 mm	14 (21.5%)	1 (1.9%)	
≥1.5 mm	10 (15.4%)	0 (0%)	
Location			
Median (range)	9 mm (0–19 mm)	0 mm (0–17 mm)	< 0.001
Category			
0 mm	14 (21.5%)	30 (56.6%)	< 0.001
1–10 mm	24 (36.9%)	17 (32.1%)	
>10 mm	27 (41.5%)	6 (11.3%)	

*SD* standard deviation

All patients underwent arthroscopy. In the ACL-injured group, a reconstruction of the ACL was performed. Most of the control patients underwent arthroscopy due to a medial or lateral meniscal tear (67.9% and 22.6%, respectively). The remaining 10% underwent arthroscopy because of chondral lesions.

### Radiological assessment

Four observers assessed all 118 radiographs and measured the depth and location of the LFNS. The median LFNS depth was 0.8 mm in ACL-injured patients and 0.0 mm in the control group (*p* = 0.008) (Table 1). The median LFNS location in relation to the Blumensaat line was 9 mm in ACL-injured patients and 0 mm in the controls (*p* < 0.001) (Table 1). The distribution of scores within the various categories for the depth and location of the LFNS was significantly different between the ACL-injured patients and the controls (both *p* < 0.001). The positive and negative predictive values (PPV and NPV, respectively) of the various cut-offs and the combined LFNS location and depth values are shown in Table 2.

**Table 2:** Positive predictive value (PPV), negative predictive value (NPV), sensitivity and specificity for different cut-off points

Cut-off values	PPV (%)	NPV (%)	Sensitivity (%)	Specificity (%)
LFNS ≥ 1.5 mm	100	49.1	15.4	100
LFNS > 1.0 mm	96.0	55.9	36.9	98.1
Location > 10 mm	81.8	55.3	41.5	88.7
Combination LFNS > 1.0 mm AND location > 10 mm OR LFNS ≥ 1.5 mm	100	53.0	27.7	100
LFNS > 1.0 OR location > 10 mm	83.3	60.5	53.9	86.8

### Inter- and intra-observer agreement

The inter-observer values for the four observers were in almost perfect agreement for the LFNS depth (0.93) and were in substantial agreement for the location (0.64). The intra-observer values for depth and location were in almost perfect agreement (0.96) and moderate agreement (0.59), respectively. The combination of sulcus location and LFNS depth was also tested for inter- and intra-observer agreement (Table 3).

**Table 3:** Inter- and intra-observer values of the LFNS depth and location between the sulcus and Blumensaat line

Measurement in mm	Single measures	95% CI	*F* (*df*1, *df*2)	*p* value
Inter-observer agreement (ICC)				
LFNS depth	0.93	0.91–0.95	54.81 (117, 351)	< 0.001
Location	0.64	0.55–0.72	8.64 (116, 348)	< 0.001
Combination of location and depth	0.80	0.75–0.85	17.54 (117, 351)	< 0.001
Intra-observer agreement (ICC)				
LFNS Depth	0.96	0.94–0.97	48.36 (117, 117)	< 0.001
Location	0.59	0.45–0.70	3.98 (117, 117)	< 0.001
Combination of location and depth	0.84	0.78–0.89	11.66 (117, 117)	< 0.001

### Correlations and associations

In the ACL-injured group, 26 patients (40%) also had a lateral meniscal injury. The median LFNS depth of patients in the ACL-injured group with lateral meniscal injury was 1.1 mm versus 0.6 mm in patients without lateral meniscal injury (*p* = 0.012). 80% of the 10 ACL-injured patients with an LFNS depth of ≥ 1.5 mm had a lateral meniscal tear compared to only 33% of patients (18 of 55 patients) with an LFNS depth < 1.5 mm, resulting in an odds ratio of 8.2 (*p* = 0.011, 95% CI 1.6–42.7). In the control group, there was no difference in LFNS depth between patients with or without lateral meniscal injury.

**Fig. 3:**
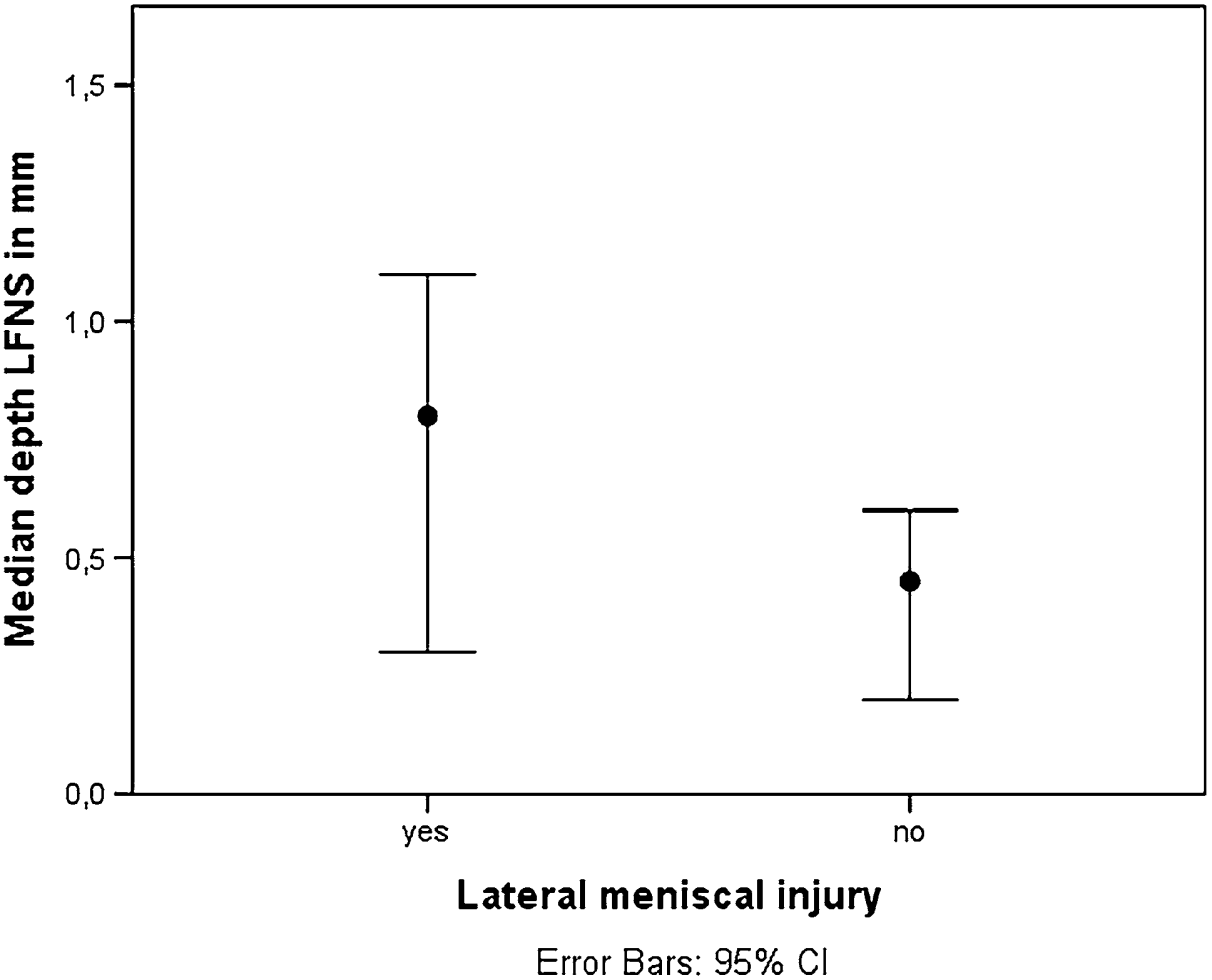
The left side shows the median lateral femoral notch sign depth in patients with lateral meniscal injury (*n* = 38), with a median depth of 0.8 mm; the right side shows the median lateral femoral notch sign depth in patients without lateral meniscal injury (*n* = 80), with a median depth of 0.5 mm (*p* = 0.040). The vertical lines show the 95% CI

In the total population (ACL-injured and controls), there were 38 lateral meniscal injuries (26 in ACL-injured patients, 12 in controls), and the median LFNS depth in lateral meniscal injury was 0.8 mm compared to 0.5 mm in the absence of lateral meniscal injury (*p* = 0.040) (Fig. 3).

## Discussion

The most important finding of the present study was that the LFNS is a clinically relevant tool for diagnosing acute ACL injuries. Furthermore, the inter-and intra-observer values showed that the depth and location of the LFNS could be reliably measured. It was postulated that a cut-off value of > 1 mm should be used in patients with acute knee injuries. When the depth of the impression of the LFNS was greater than 1 mm, an excellent positive predictive value (96%) and an acceptable negative predictive value (56%) were found. Using a higher cut-off value of 1.5 mm did not significantly increase the PPV or the NPV. However, a higher cut-off value had a substantial negative effect on the sensitivity of the test, decreasing it from 37 to 15%. The cut-off values found in this study were lower compared to those of other studies in (chronic) ACL-deficient knees [4, 9, 10, 18, 20, 26]. This could be explained by chronic instability that could result in repetitive trauma to the lateral femoral condyle and, subsequently, increased LFNS depth. The location of the LFNS, whether or not it was combined with the depth of the impression, did not contribute to the diagnostic value of the test.

The reliability of the LFNS depth measurement was very high. This was shown by high inter- and intra-observer reliability scores (ICCs of 0.93 and 0.96, respectively) [25]. The location measurement was less reproducible, with ICCs of 0.64 and 0.53 for inter- and intra-observer reliability, respectively. One of the researchers showed larger variations than the others, possibly due to inexperience in performing radiological measurements. For this reason, we recommend the sole use of the LFNS depth to predict the occurrence of ACL rupture in acute knee trauma.

In addition to diagnosing ACL injury using the LFNS depth, secondary knee pathology is more likely with a deep LFNS. Patients with lateral meniscal injury in combination with ACL injury had a significantly deeper LFNS than those without lateral meniscal injury [9, 10, 13]. This could be explained by the more forceful mechanism of trauma and impingement of the meniscus between the lateral femoral condyle and the tibial plateau.

There are studies describing the reliability of clinical examination (Lachman, anterior drawer, and pivot shift) in ACL-injured knees. Sensitivity and specificity were 0.63–0.93 and 0.55–0.99, respectively, for the Lachman test, which was described as the most reliable test in most studies [2, 14, 15, 17, 21]. Most studies did not describe clinical examination in cases of ACL injury in the acute setting. One small study found a low number of recognized ACL ruptures in the emergency department; of 27 ACL ruptures, only seven were diagnosed [7, 14]. Although this was a small study, it suggested that clinical examination was less reliable in an acute setting. A study by Katz (1986) showed a lower sensitivity of all ACL tests within 2 weeks of injury, compared to after 2 weeks [14]. The sensitivity and specificity of the LFNS in this study was comparable to those of clinical tests in the acute setting.

This study had some limitations. The study group was relatively small; nevertheless, significant differences between ACL-injured patients and controls were found. The results showed that the Blumensaat line had larger inter- and intra-observer variation [11]. Observers reported that it was often difficult to determine the beginning of the terminal sulcus. This was mostly seen in the less-experienced observer and had an effect on the inter- and intra-observer agreement of the measurement.

## Conclusion

This was the first study to investigate the inter- and intra-observer agreement of the depth and the location of the lateral femoral notch sign. The depth of the lateral femoral notch sign had a very high predictive value in ACL-injured patients and could be used in the emergency department without any additional cost. A depth of > 1.0 mm was a good predictor of ACL injury. Measuring the depth of the LFNS is a simple and clinically relevant predictor for diagnosing ACL injury in the acute setting and should be used by clinicians in patients with acute knee trauma.
